# Rediscovering Portuguese White Crowberries (*Corema album*): Cultural Insights and Nutritional Significance

**DOI:** 10.3390/foods13091328

**Published:** 2024-04-26

**Authors:** Ana Margarida Cunha, Andreia Pereira, Ana Paula Cardoso, Aida Moreira da Silva, Maria João Barroca, Raquel P. F. Guiné

**Affiliations:** 1 CI&DEI Research Centre, Polytechnic Institute of Viseu, 3504-510 Viseu, Portugal; amcunha@sc.ipv.pt (A.M.C.); arapereira@sc.ipv.pt (A.P.); a.p.cardoso@esev.ipv.pt (A.P.C.); 2 CEGOT–Centre of Studies on Geography and Spatial Planning, University of Coimbra, 3004-531 Coimbra, Portugal; 3 R&D Unit in Molecular Chemistry-Physics, Department of Chemistry, University of Coimbra, 3004-535 Coimbra, Portugal; aidams@esac.pt; 4 Polytechnic Institute of Coimbra, Coimbra College of Agriculture, Bencanta, 3045-601 Coimbra, Portugal; 5 CERNAS Research Centre, Polytechnic Institute of Viseu, 3504-510 Viseu, Portugal

**Keywords:** *Corema album*, sensorial properties, memories, emotions, valorisation

## Abstract

White crowberries (*Corema album*) are a fruit from an endemic shrub found in Southern European Atlantic costal dunes. Although this shrub and its fruits never became a formal commercial crop for a number of reasons, it has a long-lasting relevance and tradition, much associated with summer, beach and holidays. The main goal of this study was to conduct a thematic analysis of the words and small expressions people associate with white crowberries. For that, a questionnaire was used, and the participants were asked to indicate in an open-ended question which top-of-mind words/small expressions they associate with white crowberries. A total of 501 people participated in this study, of which only 394 knew about white crowberries, and from those, only 229 answered the open-ended question of interest to this purpose. The results showed that the words/small expressions given by the participants were distributed between five categories (1—Memories of places, people and times, 2—Emotions and experiences, 3—Sensorial perception, 4—Properties and uses, and 5—Natural resources’ valuation). Additionally, 18 subcategories were also identified. The most representative of the categories was sensorial perception and the most relevant of the subcategories was habitats (a subcategory from category 1). The most frequent words mentioned by the participants were beach, berry and summer. In addition, the effect of sociodemographic groups was investigated and some variations were observed in the categories of the words mentioned by the participants according to sex, living environment or region. This work allowed for the identification of a high variability in the words or expressions that account for a rich patrimony of tacit knowledge, memories, emotions and perceptions of the population towards white crowberries, thus confirming their social as well as nutritional relevance.

## 1. Introduction

Wild berries grow spontaneously in woods or mountain environments, but they are also cultivated, using either traditional or typical modern farming methods. These fruits are abundant, especially as highly coloured berries, being popularly consumed in fresh, dried or frozen forms but also in processed products such as yogurts, jams, jellies, syrups, baby foods or beverages [1,2]. Berries, especially members of several families, such as Ericaceae (blueberry and cranberry) and Rosaceae (strawberry, raspberry and blackberry) are consumed worldwide for their economic importance and numerous health benefits [1]. They have different colours, agreeable taste and flavour, low energy and are a great source of bioactive compounds that are used as functional food ingredients. Other relevant berries, though less used or applied as nutraceuticals, include lingonberries [3], cape gooseberries [4], elderberries [5], chokecherries [6], bilberries [7], cloudberries [8] and crowberries [9], among others. The bioactive compounds in berries contain mainly phenolic compounds (phenolic acids, flavonoids, such as anthocyanins and flavonols, and tannins) as well as ascorbic acid [10]. These compounds are responsible for the health benefits of berries, such as protective effects against some cancers and cardiovascular diseases and prevention of inflammatory disorders and viral infections [10,11].

**Figure 1:**
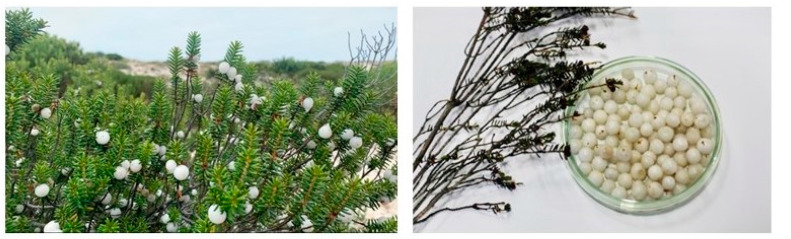
Portuguese white crowberries, *Corema album,* wild growing in sand dunes near the Atlantic Ocean, and beach pearls in a petri dish [16].

With respect to the nutritional value, berries, in general, contain a high content of sugars (glucose and fructose), dietary fibre, organic acids, some vitamins (ascorbic acid and folic acid) and low content of fat [1,10]. However, the chemical and bioactive profile of berries is affected by many factors, including variety, cultivar, ripeness stage, environmental factors, location growth and storage conditions, among others [12]. The colour of berries, associated to positive health benefits, is usually red, but some of them exhibit many shades ranging from red to black, purple, pink and yellow [13]. However, an endemic shrub found in Southern European Atlantic costal dunes, *Corema album* (L.) D. Don (Ericaceae), has white crowberries (Figure 1), also named Portuguese white crowberry, pearlberries, beachberries, Atlantic pearls or by different vernacular names depending on geographic location, such as camarina, camariña and camarinha [13,14,15].

Various theories exist regarding the euphonic origin of the name “camarina”. One theory suggests it could be a blend of the words “erica” and “marina”, reflecting its status as an Ericaceae plant that grows near the sea. Another possibility is that it derives from the name of the nymph Kamarina, a Greek deity and daughter of the god Ocean, as mentioned by Pindar and Sandys in 1915. This species holds significant cultural value in many communities along the Atlantic coast of Spain and Portugal, where its berries have long been a staple in the local diet and culture. Evidence of this cultural significance can be found in numerous streets and institutions named “Camarina”, in places like Huelva, Almonte and Moguer (Spain), as well as Tróia (Portugal) [17].

Archaeological record of the plant reveals that *C. album* has been exploited since the Early Neolithic and has been playing an important role in Iberian culture, particularly in folklore [15]. Also, according to Portuguese legend, it is said that the “camarinhas” were the tears shed by Queen Saint Isabel of Portugal upon discovering the infidelity of her husband, King D. Dinis I (13th–14th century) [18]. Additionally, “camarinhas” is a term that appears several times in the works of Gil Vicente (1465–1536), called the Trobadour, a Portuguese playwright and poet [19,20]. Recently, some popular Portuguese songs intitled Camarinhas were rescued from some ancient songbooks (“Cancioneiros”) and folklore groups and are now played by the Portuguese musical group “Toeira Trupe”. The same authors of this work identify themselves with the chapter of the book “Platero and I” by the Nobel Prize laureate in literature, Juan Ramón Jiménez (a native of Moguer (Huelva)), dedicated to the “camarinhas”, referring to them as “those edible pearls that filled my entire childhood”, as these berries have long been regarded as a delicacy, particularly by children spending summer holidays or living near the sea coast.

The plant had multifaceted uses, serving to make rustic brushes, thus explaining the origin of the genus name *Corema*, originating from the Greek word Korema, meaning broom, which alludes to its growth form [13,14,21]. Additionally, it held significance in an ancient Portuguese tradition observed during pilgrimages and religious festivities in the Gândara region of Portugal. In this tradition, young boys would present “camarinhas” to the girls they admired as tokens of affection [22].

The fruits are traditionally consumed raw but also in jellies, acid-tasting lemonades, jams or liquors, as well as used in cooking preparations or appetizers [13,14,23].

Despite its long-standing importance, this shrub and its fruits never became a formal commercial crop [14]. Factors such as climate change, urban development in coastal areas and tourism impact resulted in a deep decline of the plant in different areas of the Iberian Peninsula’s coast and a consequent decrease of fruit production and sales in traditional markets [17]. Nowadays, the sale of these berries is scarce, and its consumption is almost limited to the coast-local population and people that have memories of this fruit during the sea summer holidays [23].

These berries, that are harvested by hand from July to October, have a distinct fresh taste, with an agreeable lemony flavour and sugary and water-rich pulp (83.41–97.66%) [13,24,25]. In general, *Corema album* fruit is less sweet and more acidic than the other berries studied, presenting a total content of reducing sugars and acidity of 41.24 ± 1.74% of dry weight and 10.7 ± 0.1, expressed as g citric acid/100 g, respectively [26].

The fruits are rich in fibre (37 to 43%, dry matter) and with low levels of protein (6 to 19%, dry matter). Notably, *C. album* berries are abundant in vitamin C, providing 97 mg of ascorbic acid per 100 g. Furthermore, they exhibit a well-balanced mineral composition, with significant concentrations of potassium (121.3 mg/100 g) and calcium (21.36 mg/100 g), comparable to other berries [13,25,27].

Moreover, *C. album* has a high antioxidant activity that can be related to the content of compounds such as phenolic compounds, mainly derivatives of chlorogenic acid and flavonols, including myricetin, kaempferol and hydroxycinnamic acids, p-coumaric, caffeic, ferulic and sinapinic acid derivatives. The content of phenolic compounds, flavonols and anthocyanins in *C. album* berry extract is 77.5%, 21.8% and 0.7%, respectively, measured on a dried weight basis. The main phenolic compounds (phenolic acids) occur mainly in combined forms as esters, representing 74.2%; of which the most important are esters of caffeoylquinic acid, 5-O-caffeoylquinic acid (chlorogenic acid), 4-O-caffeoylquinic acid (cryptochlorogenic acid) and 3-O-caffeoylquinic acid (neochlorogenic acid). The free fraction is only 12.85% of the total phenolic content, meaning that the free forms failed to make a significant contribution to the composition of the total phenolic acids in *C. album* berries, with benzoic acid being the major free phenolic acid. The low content of anthocyanins in ripe *C. album* fruits can be directly linked to their white colour, as these pigments are chiefly responsible for the vibrant hues found in berries [24,28]. The presence of these bioactive compounds, associated with health benefits such as protection against oxidative stress, inflammation and cancer and prevention of neurodegenerative and cardiovascular diseases, supports the traditional use of the berries in popular medicine and suggests that the consumption of this peculiar white berry promotes human health benefits [23]. In popular medicine, the fruits have been used as an antipyretic, an antiscurvy and as a vermifuge against pinworm infections [13,15,21]. On the other hand, extracts of *C. album* leaves have the ability to prevent triple-negative breast cancer tumour progression through multiple pathways and to regulate reactive oxygen species (ROS). These effects are mainly attributed to the phenolic acids and flavonoids present in *C. album* fruit, which act as effective chemical reducers and antioxidants in cell cultures [29]. Furthermore, treatment with *C. album* extracts significantly increases cellular GSH values, the main nonenzymatic antioxidant within cells, offering protection against potential oxidative damage [21,29]. Active ingredients with reflective optical properties, such as ursolic and oleanolic acids, isolated from *C. album* berry extracts also hold particular interest in the formulation of natural sunscreens as photoprotectors [30].

However, the consumption habits of these berries are low, even in the Iberian Peninsula. Moreover, most of the population is not aware that the loss of its habitat, mainly by human actions, makes this endogenous plant of the Southern European Atlantic coast vulnerable [23]. Thus, it is crucial to increase the level of knowledge about this plant, either through the media or by intensifying scientific research to better understand the potential of this berry as functional food and to contribute to the preservation of this important biodiversity heritage of the European Atlantic coast.

The objective of this study was to conduct a thematic analysis of the words and small expressions people associate with white crowberries, an endogenous food resource from Portugal. For that, an open question asking top-of-mind words/small expressions people think of when they think of white crowberries was implemented, since this question was part of a previously used survey, published elsewhere by the group [23]. For this specific study, the expressions/words used by the participants were subject to a qualitative analysis, and a grouping structure was obtained according to the meaning of the words and their interconnection.

## 2. Materials and Methods

### 2.1. Instrument

The questionnaire used was prepared in the ambit of the project IDEAS4life and consisted of different parts as described by Moreira da Silva et al. [23]. In this work, data were used from specific parts, particularly the sociodemographic data (age, sex, education, living environment and region/interiority), answers to question about knowing what crowberries are and the open responses given by the participants to the question “Describe three to five terms or expressions that you associate to crowberries”. The questionnaire was approved by the Ethics Commission at the Polytechnic Institute of Viseu with the reference code 22/SUB/2021.

### 2.2. Data Collection

The survey was carried out on a convenience sample, according to the facility of recruitment and the disposition to answer the questionnaire, following a chain-referral sampling method for recruitment. Although aware of the limitations associated to the use of convenience samples, the benefits of recruitment and suitability for exploratory research were considered [31,32]. The snowball or chain-referral sampling strategy has been described as feasible and as a way to effectively reach otherwise hard-to-reach groups [33]. Additionally, the data collection took place during restrictions owing to the COVID-19 pandemic, more precisely between December 2019 and October 2020, and the use of internet tools and acquaintances was beneficial for recruitment. For data collection, Google Forms were used and the invitation to participate in the survey was sent through different internet tools, including e-mail and social networks. The participants had to express their informed consent before accessing the questionnaire and they could stop at any moment without submitting the answers. Additional guarantees were provided according to which the internet tool used for the questionnaire would not record personal identification data from the participants (such as email or IP address). All ethical issues were respected when designing the questionnaire and applying the survey.

The calculation of sample size, although not directly applicable for convenience samples, is a useful indicator to establish a desirable number of participants. The parameters established to estimate the sample size were a 95% confidence interval, corresponding to a level of significance of 5%, and a z score of 1.96. The power of the test considered corresponds to a minimum acceptable probability of 5% of preventing type II error [34,35]. The Portuguese population in 2020 was 10,297 thousand people [36]. Hence, considering a fraction of 80% as being adult citizens (aged ≥18 years) and establishing a target population of half of the Portuguese adults, the calculated minimum sample size was 385 participants [37,38,39].

### 2.3. Data Analysis

For the study of the relations between the sociodemographic variables and knowledge about crowberries, the chi-squared test was used, and the Cramer’s V coefficient was calculated as a measure of the association. For this, SPSS version 28 (IBM Corp., Armonk, NY, USA) was used, and a level of significance of 5% (*p* < 0.05) was considered. Cramer’s V is a coefficient varying from 0 to 1, with the following probability level: for V ≈ 0.1 the association can be considered weak, for V≈0.3 the association can be considered moderate and for V ≈ 0.5 or over, the association can be considered strong [40].

For thematic analysis, the content analysis technique was used [41]. First, a floating reading of the terms and expressions used by the participants was carried out, in order to carry out the necessary screening of responses and to constitute the documentary corpus, taking into account the rules of exhaustiveness, representativeness, homogeneity and adequacy [41]. A posteriori categorization followed, leading to the creation of five main categories that were the subject of more in-depth analysis.

Coding of the words/small expressions into the categories and then into subcategories was independently realized by two people in the software NVIVO (QSR international (2023), version 14.23.2 (46)). When in disagreement, the subcategories were discussed with a third person until an agreement was reached. The categories and subcategories were review once more by the group and some subcategories with small representation or similar themes were joined together. A total of 5 categories and 18 subcategories were created.

The translation to English of the words/small expressions was performed separately by two people. In the cases in which the translation was not consensual between the two, they discussed the translation between them until reaching an agreement. Traditional sayings and songs, as well as mentions to religious festivities, were not translated but defined as “saying”, “traditional song” and “religious festivities”.

Analysis of the impact of demographic variables in the categories/subcategories mentioned was performed in the NVIVO software and graphs were obtained through GraphPad PRISM software (GraphPad software, Inc. (Solana Beach, CA, USA). (2019), version 8.0.2 (263)).

## 3. Results

### 3.1. Sociodemographic Characterization of the Sample

A total of 501 answers to the survey were considered valid, surpassing the threshold of 385 calculated based on the assumptions mentioned earlier. From the 501 participants (Table 1), most were female (75.0%) and adults (38.5%) or senior adults (39.5%). The great majority had completed a university degree (76.8%). With respect to living place, most were from urban environments (70.5%) or from coastal areas (90.8%).

### 3.2. Associations between Knowledge about Crowberries and the Sociodemographic Variables

From the 501 participants, most answered that they knew about crowberries, more precisely 394 participants, representing 78.6%. Table 2 shows the results for the chi-square tests between knowledge about crowberries and the sociodemographic variables. No significant associations between the knowledge about white crowberries and sex (χ^2^ = 0.183, *p* = 0.669), age (χ^2^ = 3.418, *p* = 0.181) or education (χ^2^ = 1.193, *p* = 0.275) were found. On the other hand, there was a statistically significant association between knowledge about white crowberries and the area of living (χ^2^ = 10.239, *p* = 0.001). More specifically, people living in rural areas were more likely to not know about white crowberries while people in urban areas were more likely to know about them. Finally, no such association for knowledge about crowberries was observed when considering people living or not near the coast, but significance of the association was marginal (χ^2^ = 3.317, *p* = 0.069).

### 3.3. Thematic Analysis

As previously mentioned, from the 501 valid responses, 107 answered they did not know about crowberries, and, therefore, their answers to what they think about the object of study were meaningless. Additionally, from the remaining 394 participants, only 229 answered the question about writing words/small expressions they associate with white crowberries, and, consequently, only these were considered in the thematic analysis. In this question, the participants were asked to indicate up to five words or small expressions that they associate with white crowberries.

The sample for the qualitative study (*n* = 229) comprised people with ages ranging from 18 to 78 and averaging 47 (±12) years. The distribution according to the categories was 11.8% young adults (18–30 years), 43.2% adults (31–50 years) and 45.0% senior adults (>50 years). The majority of the sample participants were women (76.0%) with higher education (85.2%) living in urban areas (73.4%) close to the coast (89.1%).

**Figure 2:**
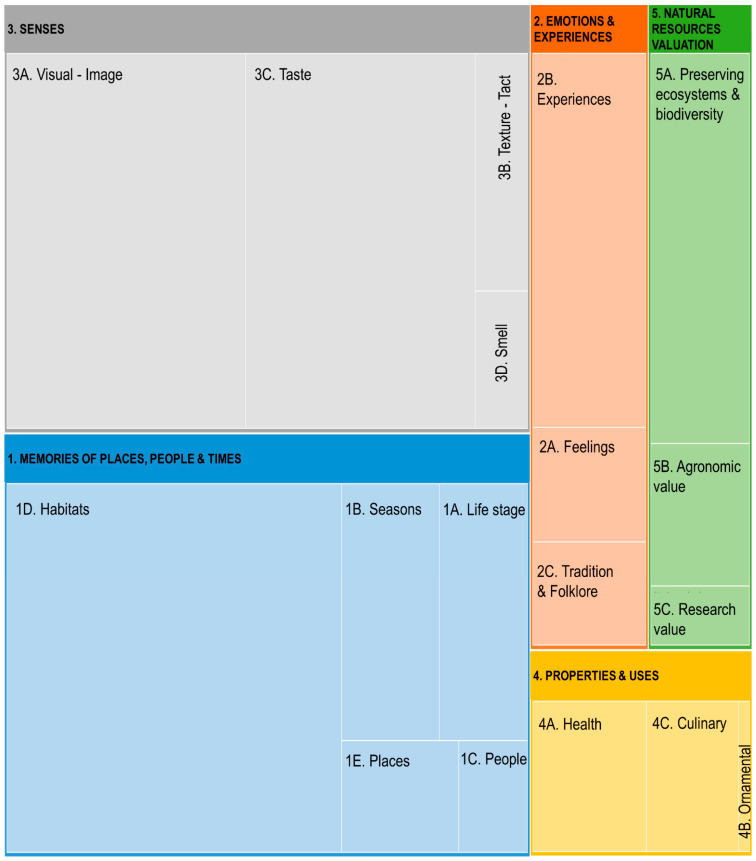
Hierarchy map of the distributions of the words/small expressions.

The inductive analysis of the words/small expressions mentioned by the participants resulted in five major categories, divided into 18 subcategories (Table 3). The categories more frequently associated with white crowberries were “Sensorial Perception” (328 references) and “Memories of places, people and times” (322 references), standing out were the subcategories “Visual image” (151 references) and “Taste” (144 references) in the first category and “Habitats” (207 references) in the second one (Figure 2).

Table 4 shows the words/small expressions used by the participants to relate to white crowberries, according to categories. A total of 925 words/small expressions were considered, of which 252 were unique.

**Figure 3:**
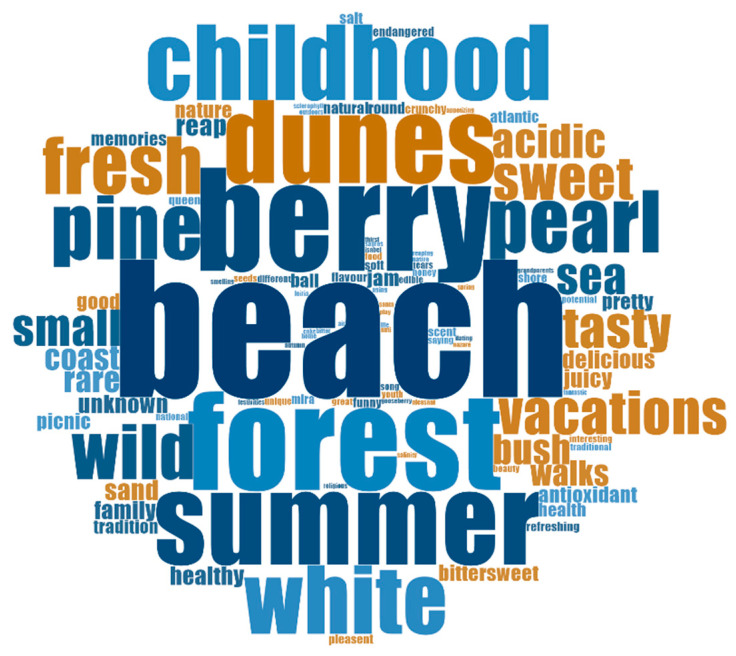
Word cloud for the whole set of words/small expressions used by the participants.

The most mentioned word was *beach* (*n* = 60), followed by *berry* (*n* = 45), *summer* (*n* = 38), *dunes* (*n* = 36) and *childhood* (*n* = 33) (Table 5). The word cloud in Figure 3 highlights the most relevant words among the multiplicity obtained in the analysis.

**Figure 4:**
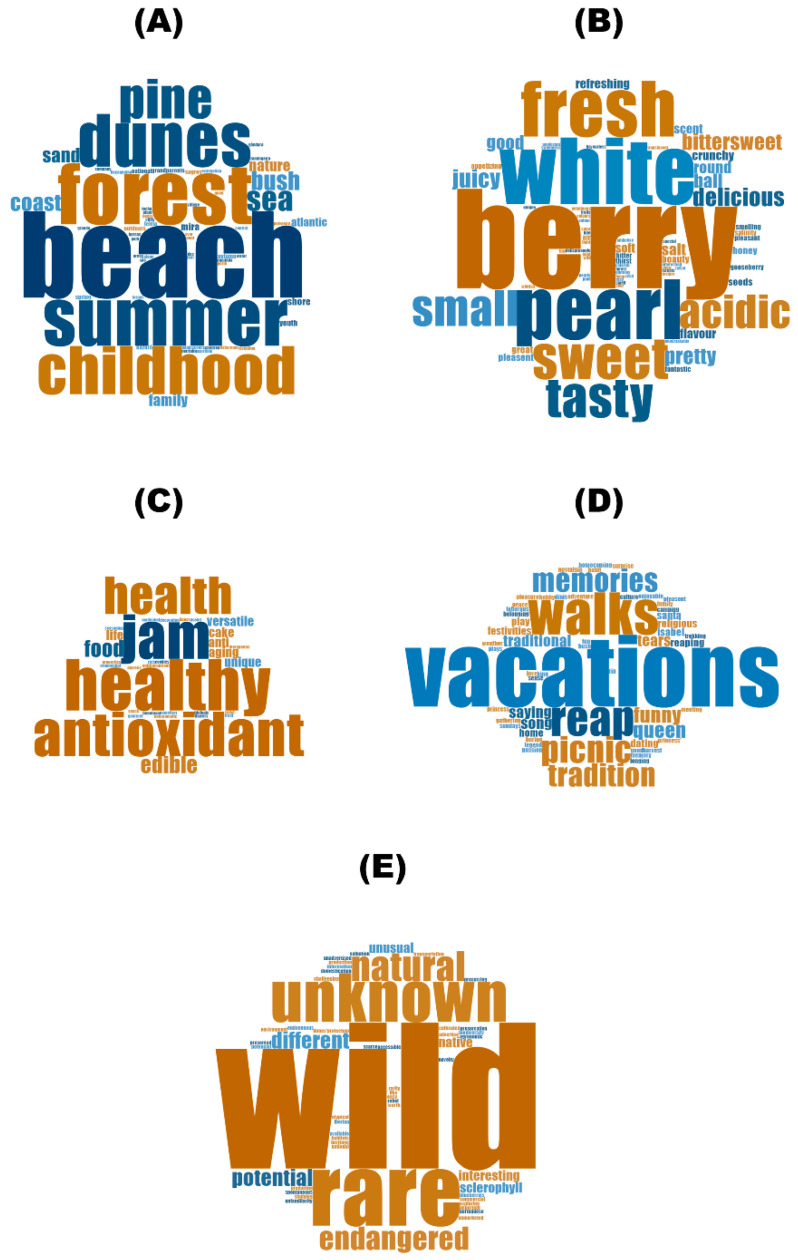
Word clouds for the words/small expressions according to categories ((**A**)—Memories of places, people and times, (**B**)—Sensorial Perception, (**C**)—Properties and uses, (**D**)—Emotions and experiences and (**E**)—Natural resources’ valuation).

When considering each category, it is possible to observe that the words present in the most common categories (“Memories of places, people and times” and “Sensorial Perception”) are the ones most mentioned in general. In that way, in the category “Memories of places, people and times” the words more referred were *beach* (*n* = 60), *summer* (*n* = 38), *dunes* (*n* = 36), *childhood* (*n* = 33) and *pine forest* (*n* = 27) (Figure 4A), and in the category “Sensorial Perception” the most common were *berry* (*n* = 45), *white* (*n* = 29), *pearl* (*n* = 27) and *fresh* (*n* = 26) (Figure 4B). In the category “Properties and uses” *healthy* (*n* = 7), *antioxidant* (*n* = 7) and *jam* (*n* = 7) were the most mentioned words (Figure 4C). Finally, in the other two categories there was a single word that was more prevalent: *vacations* (*n* = 19) for the category “Emotions and experiences” (Figure 4D) and *wild* (*n* = 23) for the category “Natural resources’ valuation” (Figure 4E).

### 3.4. Words or Expressions According to Sociodemographic Variables

**Figure 5:**
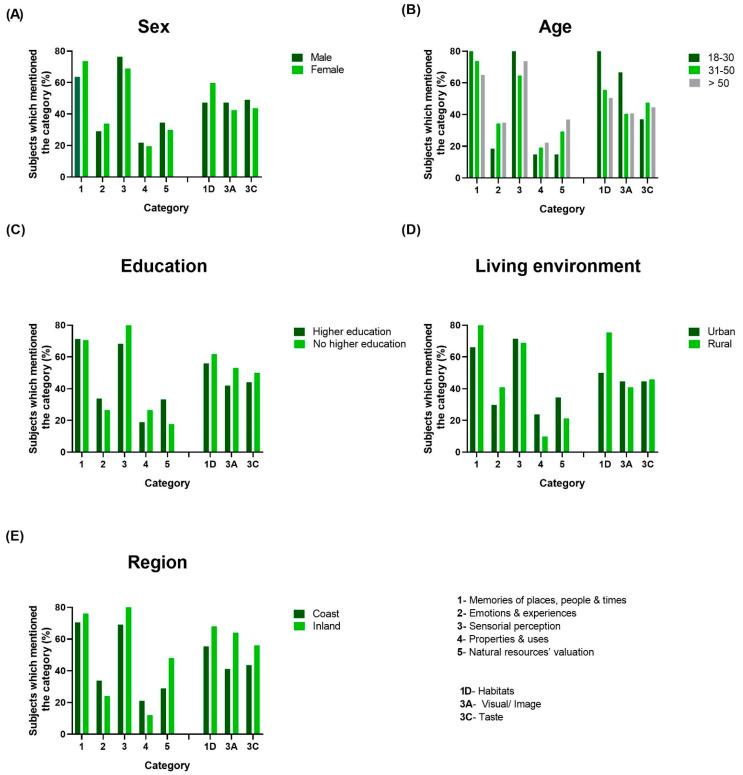
Mention of words in the five categories and the three most relevant subcategories: (**A**) sex, (**B**) age, (**C**) education, (**D**) living environment and (**E**) region.

Figure 5 shows the appearance of each of the five categories of words and the three most relevant subcategories, when they were mentioned at least once by the participants according to the different groups of the sociodemographic variables studied. These percentages were calculated as the ratio between the number of participants who mentioned the categories/subcategories in relation to the number of participants in each of the groups according to the sociodemographic variables.

With respect to sex groups (Figure 5A), no relevant differences were encountered between female and male participants. In relation to age groups (Figure 5B), some differences can be observed, especially concerning the young adults (aged 18–30 years), who mentioned the categories 2, emotions and experience; 4, properties and uses, and 5, natural resources’ valuation, less frequently than participants in other age groups, while mentioning the subcategories 1D, habitats, and 3A, visual/image, more frequently than the other participants. With respect to education (Figure 5C), very small differences were noted for all of the five categories and three subcategories evaluated. As for living environment (Figure 5D), most of the visible differences were observed between urban and rural participants in relation to the mention of words in subcategory 1D, habitats, with participants from rural environments mentioning this subcategory more frequently than those from urban areas. With respect to variable region (Figure 5E), the differences between participants from the coastal areas and those from the inland were visible, with participants from the coastal areas showing higher percentage of occurrence of words in some of the categories (2, emotions and experiences, and 4, properties and uses).

**Figure 6:**
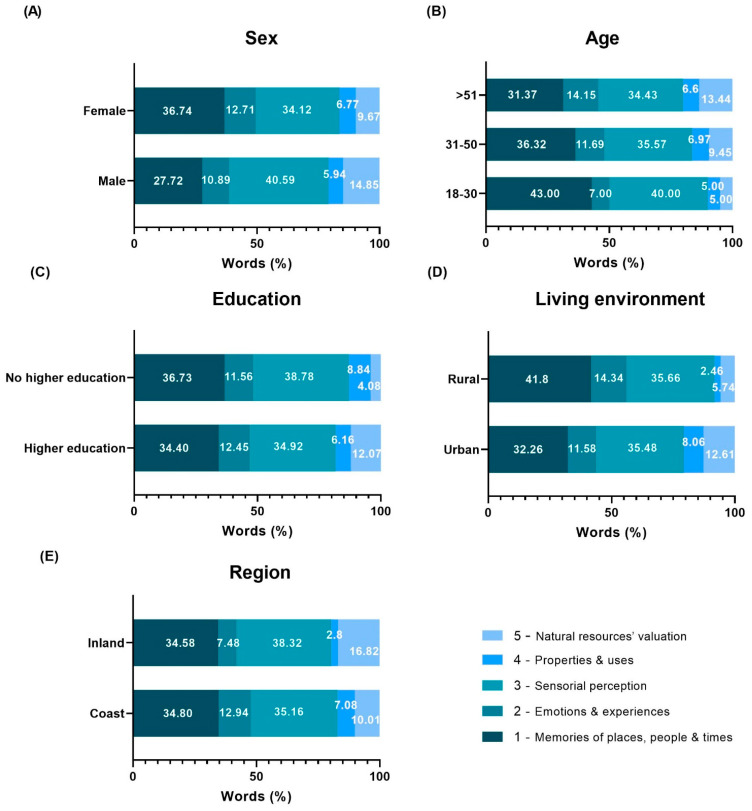
Percentage of words in each of the five categories according to the sociodemographic variables: (**A**) sex, (**B**) age, (**C**) education, (**D**) living environment and (**E**) region.

Figure 6 shows the percentage of words mentioned by the participants in each of the five categories according to the sociodemographic groups. With respect to sex (Figure 6A), while female participants mentioned more words in category 1 (memories of places, people and times), the male participants exhibited a higher percentage of words in category 3 (sensorial perception). As for the variable age group (Figure 6B), some visible differences are noted for the young adults, with a percentage of words higher for categories 1—memories of places, people and times—and 3—sensorial perception—than other age groups. When looking at the data for variable education (Figure 6C), the results are very similar in both groups (with university degree and without). As for living environment (Figure 6D), people from rural areas mentioned a higher number of words in category 1 (memories of places, people and times), while those from urban areas mentioned more words in category 5 (natural resources’ valuation). Concerning the variable region (Figure 6E), the results were very similar for participants from the coast and inland regions, with the exception of categories 4 and 5, with participants from the coastal areas mentioning more words in category 4 (properties and uses) and less words in category 5 (natural resources’ valuation).

## 4. Discussion and Perspectives for Future Work

From the analysis of participants’ knowledge and associations with white crowberries, it was confirmed that a high percentage of participants were aware of the fact that white crowberries are a type of fruit, and this knowledge is more prevalent among rural residents compared to urban dwellers [23]. This suggests that knowledge about white crowberries as a fruit is more prevalent among individuals residing in rural settings due to the proximity of the plants, especially near coastal rural settings. Studies confirm that there is a cultural heritage regarding wild plants used as food or for traditional medicine practices [42,43]. This cultural heritage of botanical species and edible wild fruits is deemed significant to preserve [44].

Using the methodology of content analysis, it was possible to investigate the words and small expressions associated with white crowberries, resulting in a classification into five categories and eighteen subcategories, with particular attention to the most representative category (sensorial perception) and the most relevant subcategory (habitats). In fact, the most relevant categories for evoking the fruit white crowberries (*Corema album*) were found to be sensory perception (visual/image, texture/tact, taste and smell) and memories of places, people and times (life stage, seasons, people, habitats, and places). This result suggests the importance of contact with nature and the value of tangible experience in understanding this fruit and its particular characteristics, whether in visual terms or in terms of texture, flavour or smell. This experience, in connection with friends, family or colleagues, allows us to create lifelong memories of places and people, which are readily remembered.

The data are in line with the perspective defended by several authors, especially Nordic ones, who defend outdoor learning and highlight the value of educational experiences in an outdoor environment, in the open air, in close proximity to natural and cultural heritage [45,46]. At a time when technologies tend to be increasingly used to access information in general, and about outdoor spaces and natural resources in particular, it is essential to encourage contact with nature and learning from outdoor activities, given the richness of sensations, emotions and experiences that this close and convivial contact awakens.

Emotions and experiences (feelings, experiences and tradition and folklore) and natural resources’ valuation (preserving ecosystems and biodiversity, agronomic value and research value) were two less-frequently mentioned dimensions, but still quite relevant, as they reinforce the importance of personal experiences in understanding this fruit in its multiple aspects (aesthetic, functional, cultural, etc.) and in valuing its potential as a natural resource to be preserved. In the case of white crowberries, this knowledge is combined with an affective/emotional relationship, an important attitudinal dimension associated with valorisation and conservation behaviours.

The least prominent dimension was properties and uses (health, ornamental and culinary). This result can be explained by the lack of in-depth knowledge of this fruit, namely its health and nutritional properties. It is therefore essential that, in parallel with experiential learning, initiatives are developed to deepen knowledge of this plant and its relevance for humans, whether in an academic context or through the media and other forms of dissemination.

The most frequently mentioned words associated with white crowberries, such as “beach”, “berry” and “summer”, shape the participants’ perceptions and experiences related to crowberries. These confirm an intrinsic relation between self-experiences and eating environments or even consumed foods. Hsu et al. [47] discuss the differences in food values across cultures and conclude that food experiences are strongly influenced by emotional value, health value and cultural value. Also, the observed variations in word categories based on sociodemographic variables, such as sex, age, education, living environment or region, reflect different perspectives, experiences and cultural influences among participants.

The results alert us to the importance of experience in the knowledge and appreciation of natural resources and in the preservation of biodiversity, essential for the desired sustainability of natural resources and, in particular, this endemic fruit of the coastal dunes of South Atlantic Europe, which in Portugal is called the Portuguese crowberry, Atlantic pearls or “camarinhas”.

This research provided insights into the memories, feelings and sensory perceptions associated with white crowberries, particularly among individuals residing in the coastal dunes of the Portuguese Atlantic coast [48]. The understanding of these associations reflects cultural, ecological or other relevant purposes. Given the discussion drawn from the research on Portuguese participants’ knowledge and associations with white crowberries, there are several potential avenues for further exploration, including dietary practices, conservation efforts, cultural traditions, economic opportunities, promotion and marketing, quality standards and certification, psychological and sociological dimensions, educational interventions and comparative studies, elaborated as follows:

1. Conservation efforts: The exploration of the implications of participants’ associations with white crowberries for conservation efforts. This could include assessing awareness levels of the ecological importance of white crowberry habitats, as well as attitudes towards conservation initiatives aimed at protecting these habitats, such as maritime pine forests and peatlands, from deforestation, pollution and other threats and their associated biodiversity;
2. Cultivation practices: Encourage sustainable cultivation practices for *Corema album* plants in suitable areas. This may involve providing guidance to farmers on optimal growing conditions, such as soil type, sunlight exposure and irrigation methods, to enhance berry yields while minimizing environmental impact;
3. Dietary practices: Investigate how the knowledge and associations with white crowberries influence dietary habits among different demographic groups. This could involve conducting surveys or interviews to understand if and how often individuals incorporate white crowberries into their diets, as well as exploring traditional recipes [13] or culinary practices involving these berries;
4. Product development: Encourage the development of innovative *Corema album*-based products, such as jams, juices, sauces and supplements, to diversify its usage and appeal to different consumer preferences. Collaborate with food manufacturers and chefs to create recipes and culinary creations that showcase the deliciousness of white crowberries;
5. Value-added processing: Explore value-added processing techniques to extend the shelf life of *Corema album* products and enhance their marketability. This may involve methods such as freezing, drying or preserving white crowberries to maintain their freshness and quality for longer periods, thereby facilitating distribution and exportation to other regions;
6. Quality standards and certification: Establish quality standards and certification schemes for *Corema album* products to ensure consistency, safety and authenticity. This instils confidence among consumers and facilitates trade by providing assurance of product quality and origin;
7. Promotion and marketing: Increase awareness of *Corema album* both locally and internationally through effective promotion and marketing campaigns. Highlight the nutritional benefits, unique flavour and culinary versatility of white crowberry to attract consumers and create demand for it in domestic and foreign markets;
8. Collaboration and networking: Foster collaboration among stakeholders in the white crowberry industry, including growers, processors, distributors, researchers, government agencies and community organizations. By working together, they can address common challenges, share knowledge and resources and implement coordinated strategies for the sustainable development of the lingonberry sector;
9. Cultural traditions: Examine the role of white crowberries in Portuguese cultural traditions and folklore. This can include the investigation of specific rituals, music, festivals or customs associated with the harvesting or consumption of these berries and how these traditions may vary across different regions or communities;
10. Psychological and sociological dimensions: Investigate the underlying psychological and sociological factors influencing individuals’ perceptions and associations with white crowberries. This could involve qualitative research methods to explore the emotional or symbolic significance of these berries within the cultural context of Portugal and Spain as well as any social norms or peer influences shaping attitudes towards them;
11. Educational interventions: Develop and evaluate educational interventions aimed at increasing awareness and knowledge of white crowberries among different demographic groups. This could involve designing educational materials, workshops or outreach programs targeted towards schools, community groups or conservation organizations [48];
12. Comparative studies: Compare the findings from our study with similar research conducted in other geographic regions or cultural contexts. This could provide insights into cross-cultural variations in knowledge, perceptions and associations with wild berries like white crowberries, as well as broader implications for biodiversity conservation and cultural heritage preservation.

By implementing these strategies, Portugal and Spain can protect the *Corema album* plant, enhance its visibility and popularity, broaden its market presence and ensure its sustainable utilization for local consumption and economic development.

## 5. Conclusions and Limitations

This work analysed the knowledge of Portuguese participants about what are white crowberries, and it also analysed words/small expressions that the participants associated with them. The results showed that a high percentage knew that white crowberries are a fruit, and this knowledge was only significantly different between participants living in urban or rural areas (with higher knowledge for people from rural areas), not varying significantly according to sex, age, education or region/interiority. The word analysis revealed five categories (the most representative being sensorial perception and memories of places, people and times) and eighteen subcategories (the most relevant being habitats). The most frequent words mentioned by the participants were beach, followed by berry and summer. Some variations were observed in the categories of the words mentioned by the participants according to sociodemographic variables, such as sex, age, living environment or region. In conclusion, this work allowed investigating memories, feelings or sensory perceptions they associate with crowberries, a wild berry growing in the coastal dunes in the Portuguese Atlantic coast.

*C. album* fruit is known by the majority of participants and continues to be highly appreciated in some regions of Portugal and Spain where this plant thrives. Therefore, enhancing the knowledge of nutritional, phytochemical and pharmaceutical properties of this singular berry alongside the sustainable exploration and fostering of its economic, cultural and gastronomic potential ensures the local consumption (raw form or as ingredients in innovative *Corema album*-based products) and fosters economic development in these regions of the Iberian Peninsula.

This work provided important insights into this endogenous product of the Portuguese Atlantic coast, from a social perspective never studied before. Nevertheless, this study has some limitations, the most relevant of which is undoubtedly related to the utilization of a convenience sample to obtain the data. Although the use of convenience samples has several advantages, like facility of recruitment, low cost and fast collection of data, it is also a fact that it does not guarantee equal representativeness of the various groups according to the sociodemographic characteristics. In this case, specifically, the sample contained more women and more graduated participants, living mostly in urban areas, while there was an under representation of young adults and people from the inland region. As so, it would be suggested as a future study to implement a similar study on a probabilistic sample, for verification.

## Figures and Tables

**Table 1:** Sociodemographic characterization of the sample (N = 501).

Variable	Group	N	%
Sex	Female	376	75.0
	Male	125	25.0
Age	Young adults (18–30 y)	110	22.0
	Adults (31–50 y)	193	38.5
	Senior adults (51+ y)	198	39.5
Education Level	Up to 12th grade (secondary)	116	23.2
	University degree	385	76.8
Living environment	Urban	353	70.5
	Rural	148	29.5
Region	Coast	455	90.8
	Inland	46	9.2

**Table 2:** Chi-square tests between knowledge and the sociodemographic variables (N = 501).

Variable	Group	Know about White Crowberries		Chi-Square Test ^1^		
		% Yes (*n* = 394)	% No (*n* = 107)	χ^2^	*p*	V
Sex	Female	21.8	78.2	0.183	0.669	0.019
	Male	20.0	80.0			
Age	Young adults (18–30 y)	24.5	75.5	3.418	0.181	0.083
	Adults (31–50 y)	17.1	82.9			
	Senior adults (51+ y)	23.7	76.3			
Education level	No university degree	25.0	75.0	1.193	0.275	0.049
	University degree	20.3	79.7			
Living environment	Urban	17.6	82.4	10.239	0.01	0.143
	Rural	30.4	69.6			
Region	Coast	22.4	77.6	3.317	0.069	0.081
	Inland	10.9	89.1			

^1^ χ^2^ = test statistic, *p* = significance (*p* < 0.05) and V = Cramer’s V.

**Table 3:** Categories of the words/small expressions used by the participants.

Group	Description	*n*	%
1	Memories of places, people and times	322	34.81
1A	Life stage	38	4.11
1B	Seasons	42	4.54
1C	People	13	1.40
1D	Habitats	207	22.38
1E	Places	22	2.38
2	Emotions and experiences	114	12.32
2A	Feelings	22	2.38
2B	Experiences	72	7.78
2C	Tradition and folklore	20	2.16
3	Sensorial perception	328	35.46
3A	Visual/Image	151	16.32
3B	Texture/Tact	21	2.27
3C	Taste	144	15.57
3D	Smell	12	1.30
4	Properties and uses	61	6.60
4A	Health	32	3.46
4B	Ornamental	3	0.32
4C	Culinary	26	2.81
5	Natural resources’ valuation	100	10.81
5A	Preserving ecosystems and biodiversity	66	7.14
5B	Agronomic value	24	2.60
5C	Research value	10	1.08

**Table 4:** Words/small expressions, according to categories.

1	Memories of places, people and times
1A	Adolescence, childhood, children, youth
1B	Autumn, spring, summer
1C	Alone, aunt Evangelina, company, family, fisherman, grandparents
1D	Atlantic, Atlantic forest, Atlantic shore, beach, bush, central coast, cliffs, coast, dunes, forest, maritime, national forest, nature, outdoors, plant, pine forest, sand, sand areas, sea, shore, Vicentina coast, water
1E	Buçaquinho park, Caminha, Fornos, Gândara, Leiria, Marinha Grande, Mira, Nazaré, Ovar, Polvoeira, Porto das Barcas, Sagres, São Pedro de Moel, Tocha, village, Zambujeira do mar
2	Emotions and experiences
2A	Boring, funny, laborious, longing, love, memories, memory, missing, nostalgia, passion, peace, pleasure, sense of belonging, surprise
2B	Adventure, camping, dating, enjoyable, family tradition, fun, gathering, good weather, habit, harvest, hobby, home, homecoming, meeting, picnic, play, plays, reap, reaping, *religious festivities*, sundays, trekking, vacations, walks
2C	Culture, King D. Dinis, legend, princess’ bush, princess’ tears, Queen Santa Isabel, queen’ tears, *saying*, tradition, *traditional song*
3	Sensorial perception
3A	Ball, beauty, berry, colourful, colour, interesting, gooseberry, pearl, pearly colour, pleasant, pretty, rosy, round, small, snow, tiny, translucent, vivid colours, white, white gooseberry, whitish
3B	Crunchy, delicate, gelatinous, juicy, light, lump, pleasant, seeds, soft, texture, thirst, velvet
3C	Acidic, addictive, appetizing, apple, bite, bitter, bittersweet, delicious, fantastic, flavour, flavourless, fresh, gluttony, good, great, honey, juicy, mild, pine forest, pleasant, refreshing, salinity, salt, soft, sour, special, sweet, sweetness, sweets, tasty, unique, unpleasant, wild
3D	Fantastic, fruity, honey scent, salt air, scent, smell, smelling, unmistakable
4	Properties and uses
4A	Antiageing, antioxidant, antitranspirant, beneficial, biologic, diuretic, health, healthy, healthy life, life, medicinal, nonedible, rich, unique therapeutic properties
4B	Decoration, flowers, ornamental
4C	Appetizer, aromatic, cake, cookies, edible, food, gourmet, jam, liquor, salad, seasoning, snack, treat, unique food, useful, versatile
5	Natural resources’ valuation
5A	Atypical, biodiversity, different, dunes protection, endangered, endogenous, environment, habitats preservation, heritage, Iberian, important, national, native, natural, Portuguese, preserving, rare, rarity, rebel, spontaneous, to be preserved, unusual, wild
5B	Accessible, advertise, agronomic potential, available, commercial, cultivated like blueberries, domestication, easy transportation, production, promoting, sclerophyll, unadvertised, unfamiliarity, unknown, unmarketed, valuation
5C	Challenging, exploring, interesting, novelty, potential, scarce information, worth studying

**Table 5:** Most frequent words/small expressions indicated by the participants.

Word	Counting	Category	Subcategory
Beach	60	Memories of places, people and times	Habitats
Berry	45	Sensorial perception	Visual/image
Summer	38	Memories of places, people and times	Seasons
Dunes	36	Memories of places, people and times	Habitats
Childhood	33	Memories of places, people and times	Life stage
White	29	Sensorial perception	Visual/image
Pine forest	27 + 1 ^(1)^	Memories of places, people and times	Habitats
Pearl	27	Sensorial perception	Visual/image
Fresh	26	Sensorial perception	Taste
Wild	23 + 1 ^(1)^	Natural resources’ valuation	Preserving ecosystems and biodiversity

^(1)^ This word appeared once in another category—Sensorial perception (subcategory taste).

## Data Availability

The original contributions presented in the study are included in the article, further inquiries can be directed to the first and corresponding authors.
